# Automatic lumen segmentation in IVOCT images using binary morphological reconstruction

**DOI:** 10.1186/1475-925X-12-78

**Published:** 2013-08-09

**Authors:** Matheus Cardoso Moraes, Diego Armando Cardona Cardenas, Sérgio Shiguemi Furuie

**Affiliations:** 1 Department of Telecommunication and Control, School of Engineering of the University of São Paulo, Av. Prof. Luciano Gualberto, Travessa 3, 158 - sala D2-06, São Paulo, SP CEP 05508-970, Brazil

**Keywords:** Intravascular optical coherence tomography (IVOCT), (IOCT), Coronary disease, Segmentation, Wavelet, Otsu, Mathematical morphology

## Abstract

**Background:**

Atherosclerosis causes millions of deaths, annually yielding billions in expenses round the world. Intravascular Optical Coherence Tomography (IVOCT) is a medical imaging modality, which displays high resolution images of coronary cross-section. Nonetheless, quantitative information can only be obtained with segmentation; consequently, more adequate diagnostics, therapies and interventions can be provided. Since it is a relatively new modality, many different segmentation methods, available in the literature for other modalities, could be successfully applied to IVOCT images, improving accuracies and uses.

**Method:**

An automatic lumen segmentation approach, based on Wavelet Transform and Mathematical Morphology, is presented. The methodology is divided into three main parts. First, the preprocessing stage attenuates and enhances undesirable and important information, respectively. Second, in the feature extraction block, wavelet is associated with an adapted version of Otsu threshold; hence, tissue information is discriminated and binarized. Finally, binary morphological reconstruction improves the binary information and constructs the binary lumen object.

**Results:**

The evaluation was carried out by segmenting 290 challenging images from human and pig coronaries, and rabbit iliac arteries; the outcomes were compared with the gold standards made by experts. The resultant accuracy was obtained: True Positive (%) = 99.29 ± 2.96, False Positive (%) = 3.69 ± 2.88, False Negative (%) = 0.71 ± 2.96, Max False Positive Distance (mm) = 0.1 ± 0.07, Max False Negative Distance (mm) = 0.06 ± 0.1.

**Conclusions:**

In conclusion, by segmenting a number of IVOCT images with various features, the proposed technique showed to be robust and more accurate than published studies; in addition, the method is completely automatic, providing a new tool for IVOCT segmentation.

## Background

Cardiovascular disease (CVD) is the number one cause of death in the United States (USA). According to the American Heart Association [1], in 2007 a rate over 2200 people lost their lives by CVD every day. It corresponded to 33.6%, more than 1/3 of all deaths. Consequently, CVD had also the greatest cost among all diseases, U$286 billion. Among CVDs, the coronary diseases are the most common, and they led to approximately 407,000 deaths in 2007, half of the CVD mortalities [1]. On account of this striking problem, equipment, tools and methods, which could lead to better diagnostic, therapies, and interventional procedure, have been attracting an enormous research interest. Consequently, the use of Intravascular medical imaging modalities, such as Intravascular Ultrasound (IVUS) and Optical Coherence Tomography (IVOCT) have become essential tools in cardiologic centers [2-5].

IVUS and IVOCT are invasive medical imaging modalities based on ultrasound and near-infrared technologies, respectively. In both modalities, image acquisition is carried out by inserting the specific catheter inside the artery and performing a pullback movement. Accordingly, cross-section images with anatomical, morphological and pathological information of arteries are provided [2,3,5,6]. As a result, more reliable diagnostic are obtained, and correct therapeutic procedure may be executed [4,7-9]. Nonetheless, only images do not supply the cardiologist with objective information, such as plaque, lumen, and elastic-lamina perimeter, radius, diameter, size, etc. [10-12]. Therefore, accurately separating related objects in an image bring special information for a range of coronary investigation; consequently, segmentation has been the scope of many studies recently [2-4,6,7,9,13].

Segmentation is a procedure in which related structures are recognized and delineated in an image, hence separating wanted object from the rest of the image [11,14]. Recognized as one of the hardest and most significant imaging processing operations, segmentation is directly or indirectly part of the great majority of imaging processing algorithm [11,15,16]. It can be executed manually by a skilled operator; semi-automatically, initialized by seed or contour and completed by an algorithm; and completely automatic, where the images are selected, and a method is applied for the entire process [17]. The implication is that, objective information of perimeter, radius, diameter, size of plaque, lumen, and elastic-lamina are supplied [11,12]. Specifically, it is important for a range of coronary investigations, for instance, quantification of stenosis, and its regression during treatment, following in-stent neointimal re-stenosis [18], and for a 3D reconstruction. As a result, diagnostic, therapy planning, treatment, evaluations, and interventional procedure are much more reliably and efficiently executed [4,11,12,19-23].

Relevant segmentation works, using a variety of methods, have been published in the last decades. The theory of Fuzzy Connectedness can be found in [11,16,24] Fuzzy applied in IVOCT was investigated in [25]. The concept of energy minimization process, dynamic programming, deformable and active contours, as well as snakes, are used in the works by [17,23,26] in which this theory is also applied in IVUS segmentation by [4,27], as well as in IVOCT by [3,28-31]. Wavelet Transformations have also a good acceptance, and have demonstrated to be a strong feature extractor in recent studies, for instance, [4,32] in IVUS, and [2] for IVOCT images. In addition, statistical and probabilistic approaches, contextual knowledge, or global image information and heuristic graph searching, gray level distribution and intensity profile analysis, can be found in [7,33,34] with IVOCT application in [35-37]. Finally, Otsu followed by mathematical morphology has been successfully applied to make binary images and post-processing them; this combination can be found in [32,38], in which they are employed in IVUS, and [29,39,40] applied similar concept in IVOCT segmentation. Specifically, [32] have successfully applied DWPF, with Otsu binarization, and Binary Morphological Reconstruction to segment the media-adventitia border and coronary wall in IVUS images.

The approaches described in the literature have used advanced and modern methodologies, and presented good results. Nonetheless, two main drawbacks can be found in most solutions. First, they are computationally demanding, due to computationally heavy operations, such as training stage. Second, they are semi-automatic, since IVUS and IVOCT are modalities that provide hundreds and even thousands of images per exam; manual, and even semi-automatic segmentation methods become a stressful and time-consuming task. In addition, they may have high variability among operators due to different initializations. Therefore, automatic segmentation methods are a more adequate and practical tool for both modalities [32,33,41]. In addition, since IVUS is older than IVOCT, engineers can find from the literature a much wider variety of methods for enhancing, or implementing solutions to create extra tools or to embed in new IVUS equipment. In order to provide this variety of methods for IVOCT as well, alternative approaches should be created, and/or successful IVUS segmentation methods, adapted and migrated to IVOCT. Therefore, a successfully applied IVUS segmentation Method, presented in [32,38], has been adequately adapted, and a new, computationally light, and fully-automatic lumen segmentation method for IVOCT images was created. A previous and concise version of this IVOCT approach was first introduced and presented in [39].

## Materials and methods

**Figure 1:**
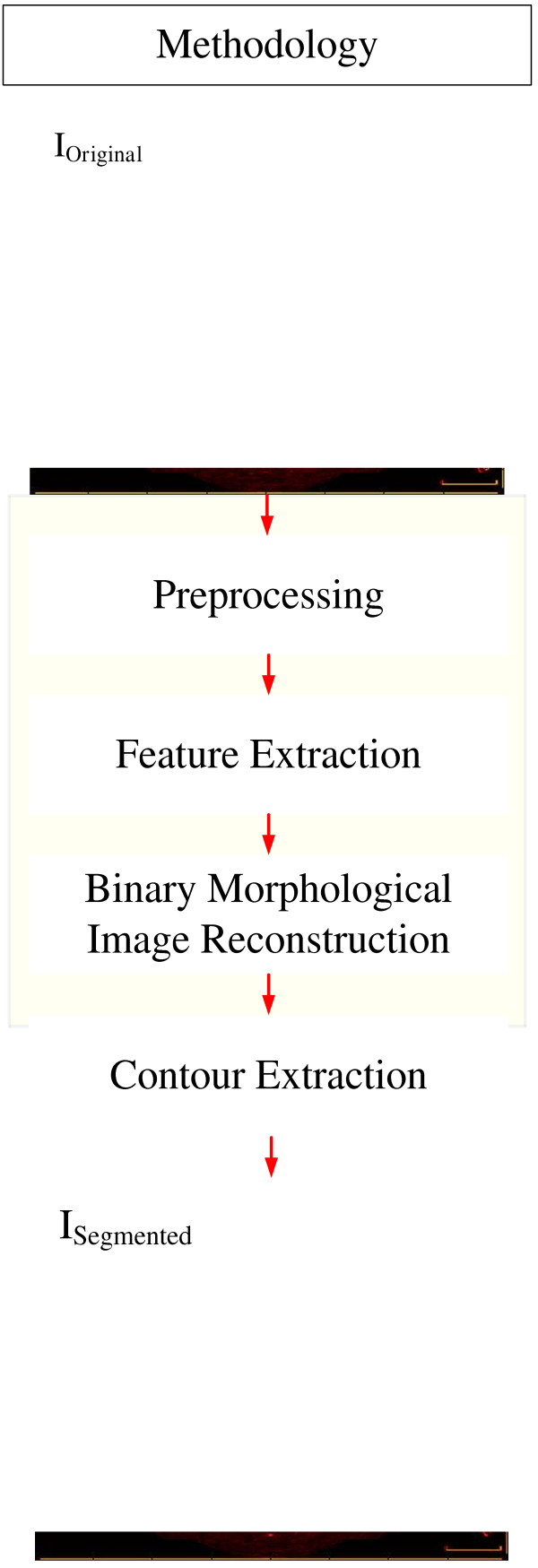
Block diagram of the major parts of the segmentation process.

The segmentation methodology is based on combining operations in three steps, Preprocessing, Feature Extraction and Binary Morphological Image Reconstruction (Figure 1). The evaluation was performed comparing the segmented images with their gold standards made by experts and calculating the parameters of accuracy [15,32]. The material is composed by a set of 290 IVOCT images from 2 patients, 2 pigs, and 1 rabbit, from the database of the Heart Institute of the University of São Paulo Clinic Hospital, Brazil (InCor). The 290 images in the dataset were chosen to represent a variety of coronary feature in IVOCT images, such as different degree of wall contrast, lumen irregularities due to thrombus, plaques and branches, and with 30 and 180 days after stent implantation, the study protocol was approved by the ethic committee of InCor with informed consent signed by patients. The images were acquired with pullback of 0.5 *mm/s*, and 20 *f/s*, by a TD-OCT, St. Jude/LightLab ImageWire catheter, connected to the St. Jude/LightLab OCT Imaging System and Probe Interface Unit (St. Jude/LightLab Optical Coherence Tomography – St. Jude Medical, Inc., Westford, Massachusetts, USA).

### Preprocessing

Because, cardiac centers have images acquired and stored in the usual format for visual evaluation, Cartesian domain with catheter reflection and alignment marks, the preprocessing block should prepares and normalizes the image, providing a standard image to the rest of the method. If OCT raw data was available, the preprocessing block could be neglected; however, since we could not ensure that all previous acquired was exported and saved in this format, the preprocessing is necessary. Therefore, beyond image normalization, this stage also aims at the attenuation and enhancement of undesirable and desirable features, respectively [4,42]. Specifically, the catheter reflection, and the alignment mark are undesirable features for this purpose, and may damage and limit the segmentation procedure. On the contrary, work with circular structures, such as coronary, in the polar domain has many advantages due to its 1D appearance [4].

**Figure 2:**
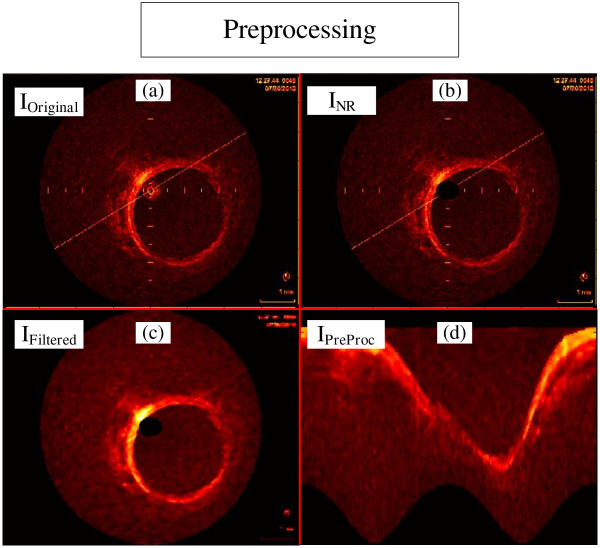
**Steps of the preprocessing stage. (a)** Original image. **(b)** Original image without catheter ring (*I*_*NR*_). **(c)** The *I*_*NR*_ image after median filtering **(d)** Preprocessed image (*I*_*PreProc*_), which correspond to *I*_*Filtered*_ in the polar domain.

The catheter reflection, and the alignment marks are recognized by a ring at the center of the IVOCT image, and straight lines marking fixed positions, and one long line crossing the image (Figure 2a). For our purpose, they can be seen as noise, hence dropping down the segmentation accuracy, because they may be misinterpreted as tissue during the Feature Extraction procedure. However, since they have known location, dimensions and characteristics, they can be removed by two simple operations. First, the catheter is removed by eliminating the concerning pixels inside the catheter ring maximum radius (*r*_*Max*_) (Figure 2b) [32]. Secondly, a 2D median filtering procedure, using 5 by 5 window, was carried out to attenuate the alignment marks, and also fading out any destructive Speckle effects without damaging borders [43,44] (*I*_*Filtered*_) (Figure 2c).

Working in an appropriate domain may help improve the method efficiency, and simplify image description [4,32]. Because the coronary has circular structure in the Cartesian image, a 1D appearance is obtained when converted to the polar domain. Therefore, so as to facilitate next procedures [7], the images were transformed into the polar representation (*I*_*PreProc*_(*r, θ*)) (Figure 2d), with *200 pixels* of *r*, equal the length of the Cartesian image radios, and *630 pixels* of *θ*, approximately equivalent to a radial variation of 0.57 degrees per line. These dimensions are important because further morphological procedures uses operations based on *I*_*PreProc*_(*200, 630*).

### Feature extraction

**Figure 3:**
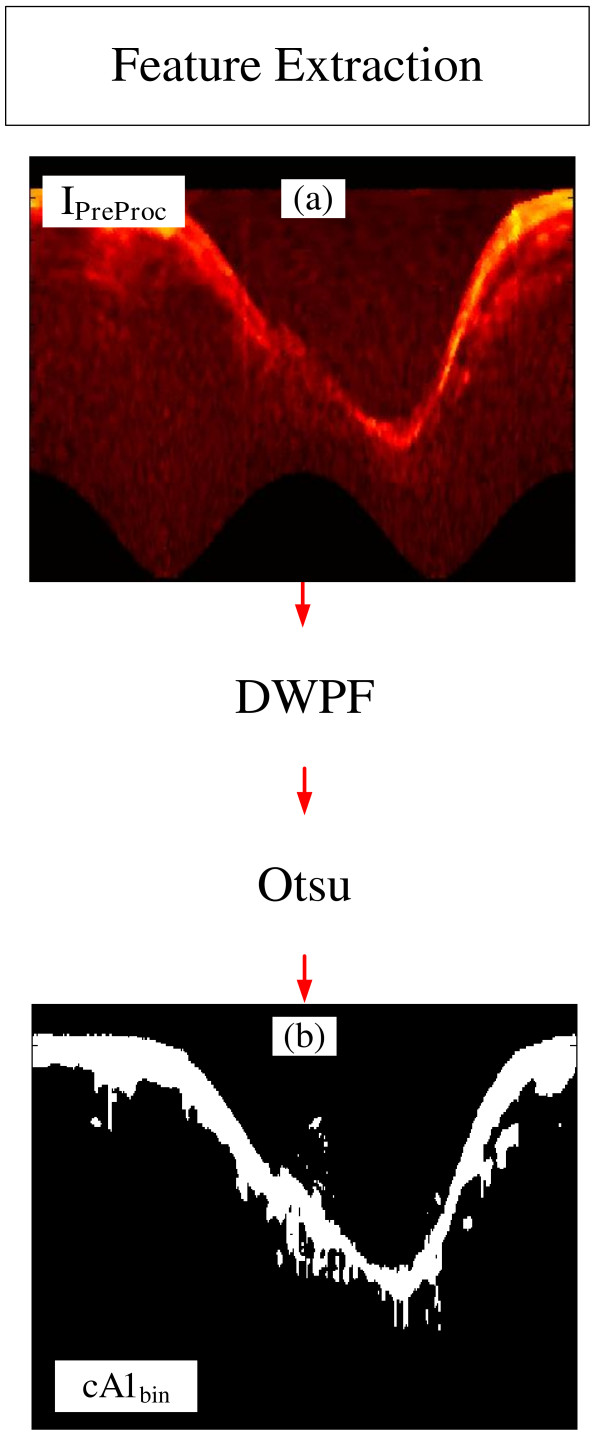
**Steps of feature extraction. (a)** Preprocessed Image. **(b)***cA1*_*bin*_, binary tissue information.

The Feature Extraction uses operations to identify and to distinguish the desired information; hence, increasing discrimination and improving classification [42,45,46]. Following what was successfully applied in [32], a combination of two widely used operations, Discrete Wavelet Packet Frame (DWPF) [42,47] and Otsu threshold [48], were adopted to acquire tissue information (Figure 3).

**Figure 4:**
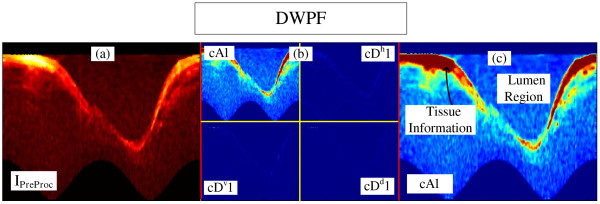
**Steps of the DWPF stage. (a)** Preprocessed Image. **(b)** DWPF coefficients of 1st decomposition. **(c)** Close look of Coefficient of Approximation 1 *cA1*.

The Discrete Wavelet Packet Frame (DWPF) is well known and has been established as a very important tool to distinguish the desired information from others, hence increasing the separability between them [4,32,42,47,49,50]. Therefore, one level of decomposition using Daubechies 1 (dB1) was carried out [32,49,50], and the *I*_*PreProc*_ image (Figure 4a) was decomposed into four coefficients (Figure 4b). The wavelet and decomposition coefficient were selected, based on the high correlation with the tissue information. As can be seen in Figure 4c, the Coefficient of Approximation 1, *cA1,* is the one that best extracted and separate tissue information (Figure 4c, between yellow to red color) from the rest of the image. Once we have the tissue information, the lumen region is directly recognized (Figure 4c). Therefore, *cA1* was chosen to be the tissue information supplier, hence serving as reference for the binary lumen object reconstruction. Binary morphological image reconstruction [32,51] is a very useful tool to estimate and polish previous information, thus increasing the method accuracy and robustness. However, a binarization process should be performed beforehand. Due to the variety of resultant IVOCT image features, according to the artery and the patient being imaged, an adaptive threshold selection is required for a good binarization.

**Figure 5:**
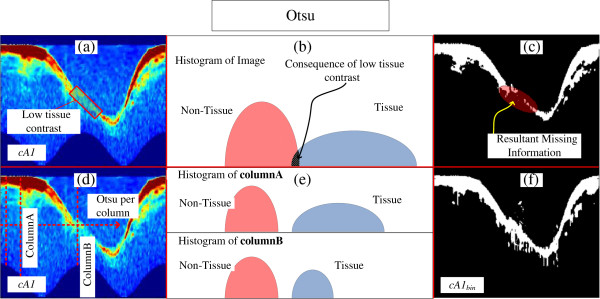
**Steps of the Otsu binarization process. (a)** Coefficient of Approximation 1 *cA1* with low tissue contrast highlighted. **(b)** Illustration of the consequent histogram of all *cA1*, with tissue and non-tissue information mixed, because of local low tissues contrast. **(c)** Resultant binary *cA1* using Otsu threshold in all *cA1;* highlighted in red is the resultant missing information due to the local low contrast. **(d)** Coefficient of Approximation 1 *cA1* with the illustration of the binarization by Otsu per column. **(e)** Illustration of the consequent histogram of columnA and B of *cA1*, since each column has tissue and non-tissue information, no matter the intensity, the histogram of each column will be bimodal, and well separated. **(f)***cA1*_*bin*_, after column Otsu binarization.

Otsu [48] is a dynamic threshold selection method for dynamic binarization process, in which a histogram is divided into two classes, by seeking for the smallest variance between two clusters, hence providing a good separation for data with bimodal histogram. Because the wavelet transformation increases the separability of desired and non-desired information, a highly bimodal histogram is created with *cA1*, which makes an adequate data to be binarized by Otsu. However, since infrared is distance sensitive, the contrast between tissue and blood may have an angular intensity variation according to the catheter location (Figure 5a, red square). Consequently, data between the two classes may appear in the histogram (Figure 5b), highlighted in black); hence, information may be lost after binarization (Figure 5c, highlighted in red). Nonetheless, the histogram of each column of the *cA1* usually has two pieces of information (Figure 5d and e), tissue and no-tissue; even when the tissue contrast is low, a bimodal histogram will be obtained (Figure 5e, column b). Therefore, we adopted a local Otsu binarization process, by column; consequently, by performing the mentioned procedure, *cA*1_bin_ is created, which corresponds to the binary version of *cA1* (Figure 5f). As a result, the Binary Morphological Reconstruction can be carried out.

### Binary morphological image reconstruction

**Figure 6:**
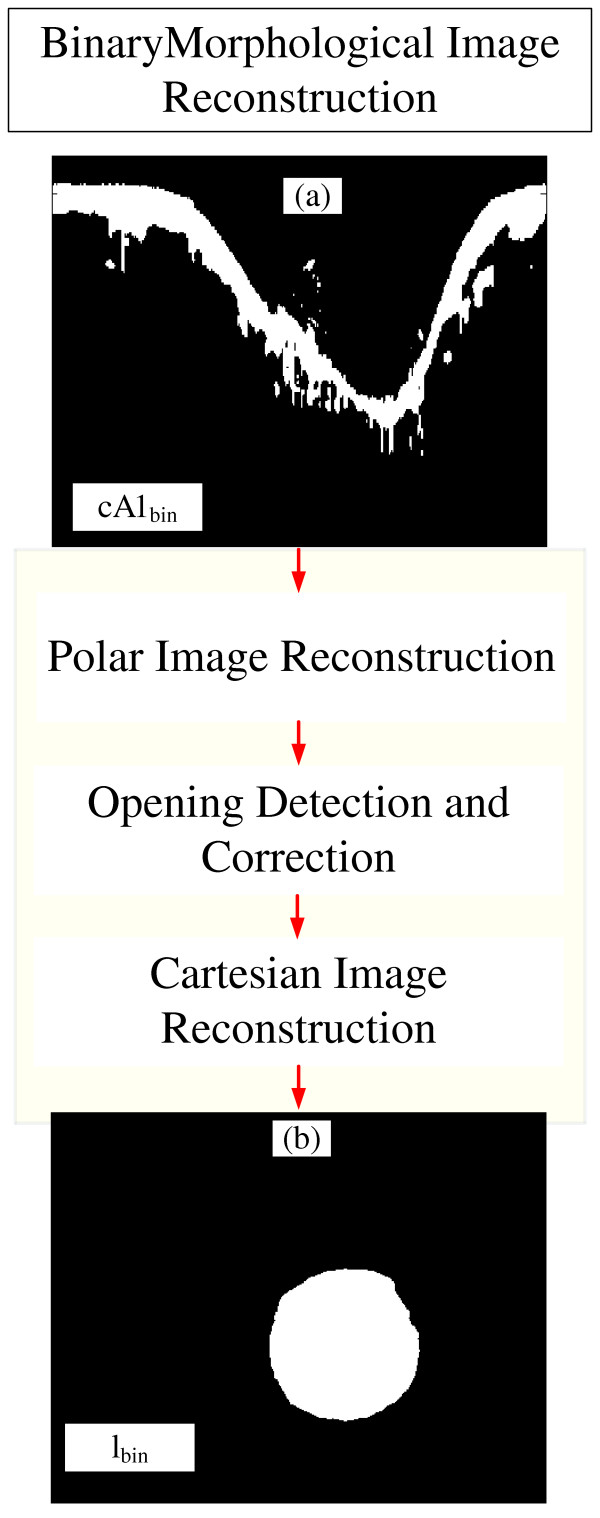
**Binary morphological reconstruction blocks. (a)***cA1*_*bin*_, the binary tissue information. **(b)** l_bin_, the binary lumen object reconstructed.

**Figure 7:**
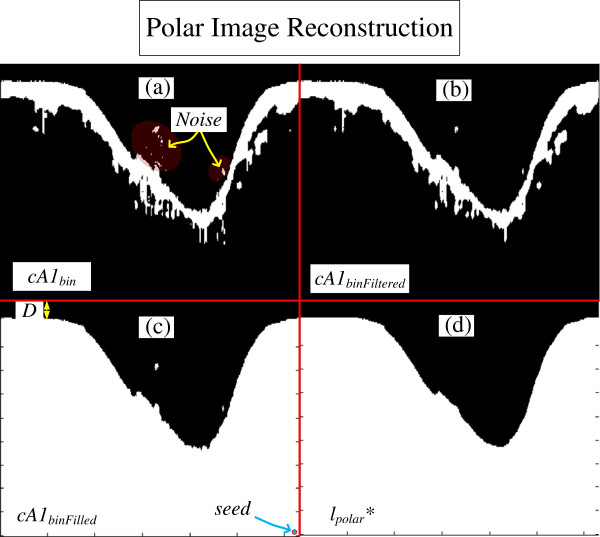
**Polar image reconstruction. (a)***cA1*_*bin*_, the binary tissue information. **(b)***cA1*_*binFiltered*_, the *cA1*_*bin*_ filtered by an opening procedure. **(c)***cA1*_*binFilled*_, is the *cA1*_*binFiltered*_, after a filling procedure. **(d)***l*_*polar*_ the complementary part of the polar lumen object reconstructed.

**Figure 8:**
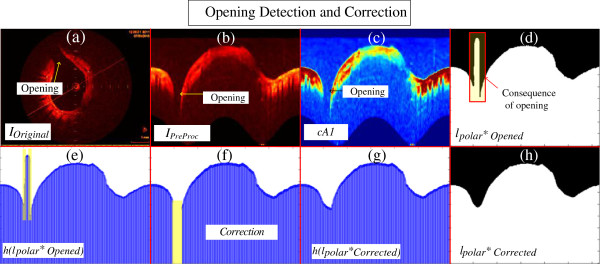
**Opening detection and correction. (a)***I*_*Original*_, Example of an Image with branch opening. **(b)***I*_*PreProc*_ consequent preprocessed image with branch opening. **(c)** Resultant *cA1.***(d)***l*_polar_*_Opened_ the polar lumen object with branch opening. **(e)** Height of the final polar image represented by signal *h(l*_polar_*_Opened_), high derivatives highlighted in yellow, indicates the presence of opening*.***(f)***h(l*_polar_*_Opened_*)* with correction being performed, values corresponding to the opening are removed for interpolation. **(g)***h(l*_polar_*_Corrected_*)*, Signal representing the column corrected. **(h)** The corrected polar lumen object *l*_polar_*_Corrected_.

**Figure 9:**
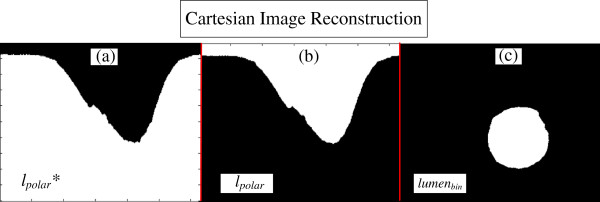
**Cartesian image reconstruction. (a)***l*_polar_* the complementary part of the polar lumen object reconstructed. **(b)***l*_*polar*_, the polar lumen object reconstructed. **(c)***lumen*_*bin*_, final binary lumen object reconstructed in the Cartesian Domain.

Binary morphological Image reconstruction is a sequence of combined mathematical morphology techniques [32,51,52] designed to obtain an accurate binary version of the desired object. Particularly in this block, we used the previous information, *cA1*_*bin*_ (Figure 6a), to obtain the corresponding binary lumen object, *l*_*bin*_ (Figure 6b). In order to accomplish this task, the operations for the reconstruction are divided into three parts, Polar Image Reconstruction, Opening Detection and Correction, and Cartesian Image Reconstruction (Figure 6). The first, polar image reconstruction, aims to obtain the complete complementary part of the polar lumen object, *l*_polar_*, (Figure 7). If the image has branch opening, the opening detection and correction block is performed to correct it (Figure 8). The final binary lumen object, in the Cartesian domain, is reconstructed during the Cartesian image reconstruction (Figure 9). Each block is detailed below:

### Polar image reconstruction

Polar Image Reconstruction is a combination of binary morphological procedure applied in the polar domain information, *cA1*_*bin*_, so that it can be refined, and possible missing information estimated (Figure 7). Due to the range of artery and blood features of patients, spurious noises may appear in a variety of sizes and quantity in the lumen region in the *cA1*_*bin*_ (Figure 7a). Since they could also be connected to the tissue information, these noises must be removed. To remove them, we first disconnect them from the main tissue information block by filtering the image with a morphological opening procedure [51,52] resulting in *cA1*_*binFiltered*_ (Figure 7b); second, an upward filling procedure [32,51-53], resulting in *cA1*_*binFilled*_ (Figure 7c), followed by an area selection generating the *selected*(*cA1*_*binFilled*_) is carried out; finally, a last closing procedure [51,52] is performed, obtaining the complementary polar lumen object, *l*_polar_* (Figure 7d). The opening and closing procedures uses circular structuring elements, with 3-pixel and *D* pixels diameters, respectively. The circular elements is to maintain the smooth contour of object, and *D = r*_*Max*_ pixels, is an adaptive diameter where *r*_*Max*_ correspond to the catheter ring maximum radius [32]. This size assures that possible lumen border irregularities will be attenuated without changing original contour or connecting the object to top of the polar image.

### Opening detection and correction

Branch openings are shadows in IVOCT images caused by vessel bifurcations during image acquisition (Figure 8a) [32]. Consequently, they are propagated to the preprocessed image and *cA1* (Figures 8b, and c). Because the gap does not produce contrast in its columns, a bi-modal histogram is not generated; thus, the columns corresponding to the gap are binarized as level “1” (*l*_polar_*_Opened_) (Figure 8d). This causes high derivative at the lumen border shape of the *l*_polar_*_Opened_ (Figure 8d). Therefore, its detection and correction is carried out as follows. First, a signal representation of the polar image is created *h(l*_polar_*_Opened_) (Figure 8e). Second, the signal derivative is calculated, and by finding values higher than a threshold, the opening is detected. Consequently, the correction initiates by removing all the values corresponding to the gap (Figure 8f), and performing Piecewise cubic Hermite interpolation (Figure 8g). Finally, the corrected polar image (*l*_polar_*_Corrected_) (Figure 8h) is then reconstructed using the interpolated signal. The beginning and end of the gap are identified as the first and last derivative absolute values, respectively higher than the threshold, which is defined as *5 standard deviation* of all derivative signals.

### Cartesian image reconstruction

**Figure 10:**
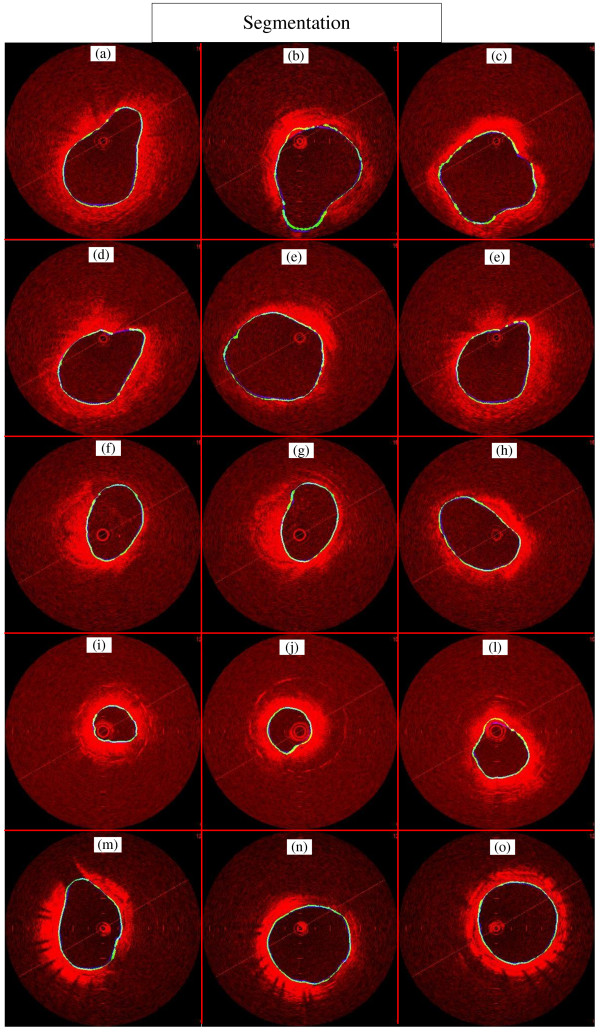
**Segmentation outcomes.** The blue line is the gold standard, and the green one is the contour made by this approach. The images from human and pig coronaries, and rabbit iliac arteries have different level of tissue contrast, lumen irregularities due to thrombus, plaques and branches, with stent after 30 and 180 days of implantation.

The Cartesian image reconstruction combines an image domain transformation with one last morphological operation, for object polishing. Therefore, *l*_polar_* (Figure 9a) is obtained, no matter if it went through the opening correction. First the logic negation of *l*_polar_* is carried out; hence, obtaining the lumen object in the polar domain, *I*_*polar*_ (Figure 9b). Finally, the lumen reconstruction, *lumen*_*bin*_ (Figure 9c), is concluded by transforming to the Cartesian domain, *I*_*Cartesian*_ followed by one last opening operation [51,52], with a circular structuring element with adaptive diameter *Rmin* pixels (*S*_*circ(Rmin)*_), where *Rmin* is the minimum radius between the center and border of the lumen. This last opening is because possible irregularities in the polar domain will be carried to Cartesian. Using a circular element, these remaining irregularities are removed, and a smooth contour of the object is obtained. Finally, the segmentation is concluded by extracting and placing contour of *lumen*_*bin*_ on the Original image [52] (Figure 10).

## Results and discussion

The proposed approach was evaluated by segmenting and computing the parameters of accuracy in 290 IVOCT challenges images, which experts established gold standards for the lumen. The database was composed of images with different vessel size and features, such as irregularities and eccentricity of lumen due to thrombus; plaques; branches; several tissue contrasts, and stent implanted 30 and 180 days before acquisition (Figure 10). The image segmentation was performed in a Desktop computer with an Intel Core 2 Duo 2.53 GHz, 4 GB of RAM, Windows Vista 32 bits and MATLAB (2009a) without code optimization. The average time of the lumen segmentation, using the software and computer described above, was (5.9 ± 3)*s*; apart of being faster than manual segmentation, which is above one minute per image, it is more practical and much less exhaustive, since hundreds of images are provided. Code optimization or the use of other computer language, such as C++ or Java certainly may improve even more the processing time.

### Assessment of accuracy

The accuracy was obtained by computing from the 290 images the average and standard deviation of the following parameters: True Positive Area Fraction (*TP*), the False Positive Area Fraction (*FP*), the False Negative Area Fraction (*FN*), as well as the Maximum False Positive Deviation (*Max*_*FP*_), the Maximum False Negative Deviation (*Max*_*FN*_). Figure 10 shows a sample of the segmented images and their accuracy. The good accuracy can be verified in Table 1, in which the *TP* yielded more than 99% of agreement, and *FP* slightly higher than 3%; the method precision and robustness can be seen by the small standard deviation of the indexes a lower than 3% (Table 1), and the small *Max*_*FP*_, and *Max*_*FN*_, with average smaller than 0.1 *mm* in both indexes, for an image size of 6 *mm* × 6 *mm.*

**Table 1:** **Assessment of accuracy **[15]

		**Parameters**				
***T P***	***F P***	***F N***	*** Max***_***FP***_	***Max***_***F N***_	***OR***	***OD***
**(%)**	**(%)**	**(%)**	**( *mm *)**	**( *mm *)**	**(%)**	**(%)**
99.29	3.69	0.71	0.1	0.06	95.4	97.8
±2.96	±2.88	±2.96	±0.07	±0.1	±4.8	±2.16

Pixel size: 15 *μm* x 15 *μm.*

Image size: *400 x 400 pixels.*

Conclusions among different methods should be carried out comparing results using the same database, computer, and software. Therefore a direct comparison among published methods and this approach is not in the scope of this paper. Nonetheless, pointing out equivalences between published and new methods may be useful to support comparisons. The method efficiency, high accuracy, precision, and robustness were corroborated by computing and comparing the related parameters (Table 1) to equivalent works. The computational cost provided in the proposed method, approximately *6s* per frame, is in line with the one proposed by [36], which makes 100 frames of stent-IVOCT segmentation in *15 min*. The works presented by [30,31] obtained costs of less than *1s* per image using C++. In order to compare our accuracy to other methods, the Overlap Ratio (OR) [2] and Overlap Dice (OD) [3] were also computed, values close to 96%, and 98%, were obtained respectively (Table 1). In [2], their lumen segmentation approach presented an OR near 94%. In the paper presented by [3], an appreciable value of *OD* close to 97% was obtained. In the semi-automatic method presented in [25] led to results close to 96% and 98% of *OR* and *OD,* respectively. As a result, our outcome accuracy rendered efficacy as high as results from mentioned works [2,3,25]. The proposed method has the advantage of being completely automatic and also composed by operations known to be simpler and lighter, in which the use of heavy computational tasks, related to energy minimization procedures, were prevented.

The Fourier-Domain OCT technology (FD-OCT) has rapidly increasing its use among cardiologists, and it is currently considered a better choice for IVOCT images. However, since time-domain OCT technology (TD-OCT) is the only available solution in many locations, it is still a useful tool, fulfilling most of the requests of clinics and hospitals, such as follow-up in stent neointimal re-stenosis [18]; hence, it may not be completely replaced very soon. Therefore, tools and methods dedicated to automate TD-OCT image applications are still useful. During the development of the proposed method, the TD-OCT was the only available choice, kindly supported by our collaborator (InCor). Using images from others sources, additional ethics protocols and new collaboration policies should have been established. Due to that, the current methodology was fully created based on TD-OCT technology and its image features.

Indeed, because different IVOCT technologies, for instance the TD-OCT and FD-OCT, provide a different image texture, the Feature Extraction and Morphological Operations blocks of this methodology would have to be modified so as to work in both technologies. However it is not in the scope of this work. Therefore, efforts will be made to access FD-OCT technology by additional collaborators and partners; consequently, this method will be adapted to work in both technologies. Beyond that, futures works will investigate techniques to create an alternative stent segmentation method; hence, permitting a 2D neo-intima re-stenosis quantification. Additionally, solutions to overcome the challenges of an accurate artery 3D reconstruction will be pursued; hence, providing complete volumetric artery information, speeding up investigation and bring more details to follow in-stent neointimal re-stenosis.

## Conclusions

The importance of IVUS and IVOCT segmentation, to directly or indirectly contribute to numerous investigations, is a topic of great concern in many research groups [2-4,27-32,38]. The mentioned papers have provided a variety of interesting methods and good results. Nonetheless, a method that gathers the best of different features such as accuracy, practicability, and good computational demand is still on track. Consequently, new and alternative approaches which could improve one or some of the features are very welcome.

We presented an alternative methodology, combining wavelet and mathematical morphology. This methodology was successfully employed in previous studies [32,38], and was now adapted and applied for the lumen segmentation in IVOCT (TD - OCT) images. The methodology was based on four stages. The first, Preprocessing block, the image is prepared, and normalization and filtering are carried out. The second, Feature Extraction, tissue information is obtained by Wavelet transform and Otsu [48]. Next, Binary Morphological Image Reconstruction is performed. Finally, the segmentation is concluded via Contour Extraction**.**

Good efficiency, accuracy, computational cost and practicability have motivated the development of the proposed method for IVOCT images. The major specific contributions are: (a) A wavelet associated with an alternative version of Otsu for tissue information extraction, and; (b) a new sequence of morphological operations, designed to reconstruct lumen object, resulting in an accurate segmentation, even in the presence of bifurcation structures.

## Abbreviations

IVOCT: Intravascular optical coherence tomography; IVUS: Intravascular ultrasound; CVD: Cardiovascular disease; InCor: Heart institute of the University of São Paulo Clinic Hospital; TP: True positive; FP: False positive; FN: False negative; MaxFP: Max false positive distance; MaxFN: Max false negative distance; OR: Overlap ratio; OD: Overlap dice; DWPF: Discrete wavelet packet frame.

## Competing interests

Other than the grants listed in the acknowledgement section, the authors declare that they have no other competing interests.

## Authors’ contributions

All the authors contributed to the manuscript creation. MCM designed the investigation, created the methodology and carried out the evaluation, data analysis and the manuscript. DACC contributed to the design of the investigation, methodology, literature revision, data analysis and result interpretation. SSF coordinated the entire work, designed the study and evaluation, managed the interaction with medical doctors, supervised and critically revised the manuscript creation, giving substantial suggestions. All authors read and approved the final manuscript.
